# Characterization of an *Escherichia coli* ST156 Isolate Harboring an IncHI2-Type Plasmid Co-Carrying *bla*_NDM-5_ and *mcr-1.1* Genes from Urban Wastewater Treatment Plants in Fengxian, Shanghai

**DOI:** 10.3390/antibiotics15030275

**Published:** 2026-03-06

**Authors:** Qingyuan Zhang, Xiaohong Xie, Lixin Tao, Jian Wang, Yuan Shi, Huangfei Sheng, Chuanlong Liu, Hongwei Zhao, Meihua Liu, Jun Feng

**Affiliations:** 1 Shanghai Fengxian Center for Disease Control and Prevention, Shanghai Fengxian Health Supervision Institute, Shanghai 201499, China; qingyuanzhang0109@126.com (Q.Z.); xxh6611@sina.com (X.X.); 18001600436@163.com (L.T.); fxcdchuanwei@163.com (J.W.); shiyuan15221@163.com (Y.S.); 15821160368@163.com (H.S.); liuchuanlongx@163.com (C.L.); 2 Shanghai Municipal Center for Disease Control and Prevention, Shanghai 201107, China

**Keywords:** *mcr-1.1*, IncHI2, *bla*_NDM-5_, urban WWTPs, *Escherichia coli*

## Abstract

**Background**: The emergence of carbapenem-resistant enterobacteriaceae (CRE) co-harboring the *mcr-1.1* gene and carbapenemase-encoding genes poses a severe threat to public health. Urban wastewater treatment plants (WWTPs) act as natural reservoirs and hotspots for the dissemination of antimicrobial resistance genes (ARGs). This study aimed to elucidate the molecular characteristics of CRE carrying *mcr-1.1* in urban WWTPs. **Methods**: Samples were collected from the influent of urban WWTPs in Fengxian, Shanghai, from April 2024 to March 2025. *mcr-1.1*-positive *Escherichia coli (E. coli)* isolates were screened using real-time PCR, and their antimicrobial susceptibility was determined via the broth microdilution method. Plasmid conjugation assays were performed with *E. coli C600* as the recipient strain. Whole-genome sequencing (WGS) was carried out to analyze the molecular characteristics of *mcr-1.1*-positive *E. coli* isolates. **Results**: A total of 312 samples were collected, and 5 (1.6%) *mcr-1.1*-positive *E. coli* isolates were identified. All isolates were multidrug-resistant (MDR) but susceptible to tigecycline (TIG). WGS of strain EC0176 (sequence type 156 [ST156], enteroaggregative *E. coli* [EAEC]) detected the presence of *bla*_NDM-5_*, bla*_TEM-*1*_*, bla*_CTX-M-55_, and *mcr-1.1* as well as related virulence genes. Further analysis revealed that pEC0176 was an IncHI2-type plasmid co-harboring *mcr-1.1, bla*_NDM-5_*, arr-3, aph(4)-Ia, aph(3′)-Ia, aac(3)-IVa*, and *mph(A)*. The plasmid pEC0176 harbored similar backbones as p20014-MCR, p2017.03.02CC_1, pSC2017167-mcr-256k, pEC17CM13_MCR and pGDE043-mcr1, including the type IV secretion system (T4SS) and IncHI-type conjugal transfer genes. Conjugation experiments confirmed that pEC0176 could be horizontally transferred into *E. coli* C600, with an average transfer efficiency of 3.3 × 10^−2^. Phylogenetic analysis showed that the MCR-1 protein of EC0176 is closely related to that of two human-derived *E. coli* strains from China (GenBank accession: AVR64822.1 and WP_076611062.1). **Conclusions**: To our knowledge, this is the first report of *E. coli* ST156 carrying an IncHI2-type plasmid co-harboring *mcr-1.1* and *bla*_NDM-*5*_ from urban WWTPs in Fengxian, Shanghai. Our findings underscore the severe status of bacterial antimicrobial resistance and emphasize the necessity of enhancing antimicrobial resistance surveillance in urban WWTPs.

## 1. Introduction

The escalating prevalence of CRE poses a severe threat to human and animal health, as well as to the ecological environment [1,2]. Colistin has long been regarded as the “last-resort” antimicrobial for the treatment of CRE infections [3]. However, the discovery of the plasmid-mediated colistin resistance gene mobile colistin resistance-1 (*mcr-1*) in 2015, initially identified in animal samples, retail meat products, and human clinical specimens in China, has significantly compromised the therapeutic options for CRE infections [4]. The coexistence of *mcr-1* with carbapenemase-encoding genes, such as those producing the New Delhi metallo-β-lactamase (NDM), can lead to the emergence of MDR or even pan-drug-resistant pathogens, which has raised substantial clinical concern [4].

Since the first report of NDM-producing *E. coli* in China in 2014, NDM-*5* has rapidly become the predominant variant, exhibiting higher resistance to carbapenems and extended-spectrum cephalosporins compared with NDM-1 [5,6,7]. Plasmid-mediated conjugative transfer constitutes a major mechanism driving the dissemination of ARGs among bacterial populations. The *mcr-1* is carried by various plasmid types, with IncI2, IncX4, and IncHI2 being the most prevalent [8,9,10,11]. Furthermore, the association of *mcr-1* with different composite transposons can enhance its stability and transferability, facilitating its persistence in bacterial populations [12].

To date, *E. coli* strains co-harboring *mcr-1* and *bla*_NDM-5_ have been reported in multiple regions, predominantly in clinical and animal samples [13,14,15,16,17,18]. However, ARGs can spread across various environmental compartments via their microbial hosts [19]. Aquatic environments are recognized as important reservoirs and transmission routes for ARGs, enabling their long-distance dissemination [20]. Urban WWTPs receive a complex mixture of domestic sewage, livestock wastewater, agricultural runoff, initial rainwater, and surface water, thereby serving as key reservoirs for ARGs mobilization [21,22]. Evidence indicates a strong correlation between ARGs and their microbial hosts in sewage, and wastewater is recognized as a hotspot for horizontal gene transfer [23,24]. These findings underscore the high risk of ARGs persistence and colonization in urban aquatic environments, emphasizing the urgent need for systematic monitoring.

In China, surveillance of *mcr-1*-harboring CRE in urban wastewater remains limited [25,26]. Given this evidence gap, we conducted a genomic investigation to characterize the antimicrobial resistance profiles, plasmid contexts, and phylogenetic relationships of *mcr-1*-positive *E. coli* isolated from the influent of urban WWTPs in Fengxian, Shanghai.

## 2. Results

### 2.1. Identification of Sewage Samples

Between 1 April 2024 and 31 March 2025, a total of 312 samples were collected from urban WWTPs in Fengxian, Shanghai. Of these samples, five (1.6%) *mcr-1.1*-positive strains were isolated that carry the *mcr-1.1*, the *mcr-1.1-*positive strains in this study were mainly concentrated in summer and winter: two were obtained from the WWTP-W (EC0175, EC0176), three from WWTP-E (EC0177, EC0178, EC0179). Further species identification revealed that all five strains were *E. coli*, with one strain (EC0176) identified as EAEC (Table 1).

### 2.2. Antimicrobial Susceptibility Testing

All five isolates were identified as MDR strains, with each exhibiting a distinct antimicrobial resistance profile. Strain EC0176 was resistant to 25 antimicrobial agents, with an multidrug resistance index (MDRI) of 0.93 (Table 2). The minimum inhibitory concentrations (MICs) and corresponding ratings of resistant (R), intermediate (I), and susceptible (S) of the five strains harboring *mcr-1.1* are listed in Table 3. All strains were uniformly resistant to florfenicol (FFC), ampicillin (AMP), and cefazolin (CFZ). In addition, these strains exhibited high-level resistance to most cephalosporins and quinolones, while all were susceptible to tigecycline (TIG). The MICs of colistin against all five strains were 4 μg/mL. Specifically, the MIC values of strain EC0175 against colistin (COL) and polymyxin B (POL) were >8μg/mL and >4μg/mL, respectively (Table 3).

### 2.3. Molecular Features of mcr-1.1-Positive Strains

**Figure 1:**
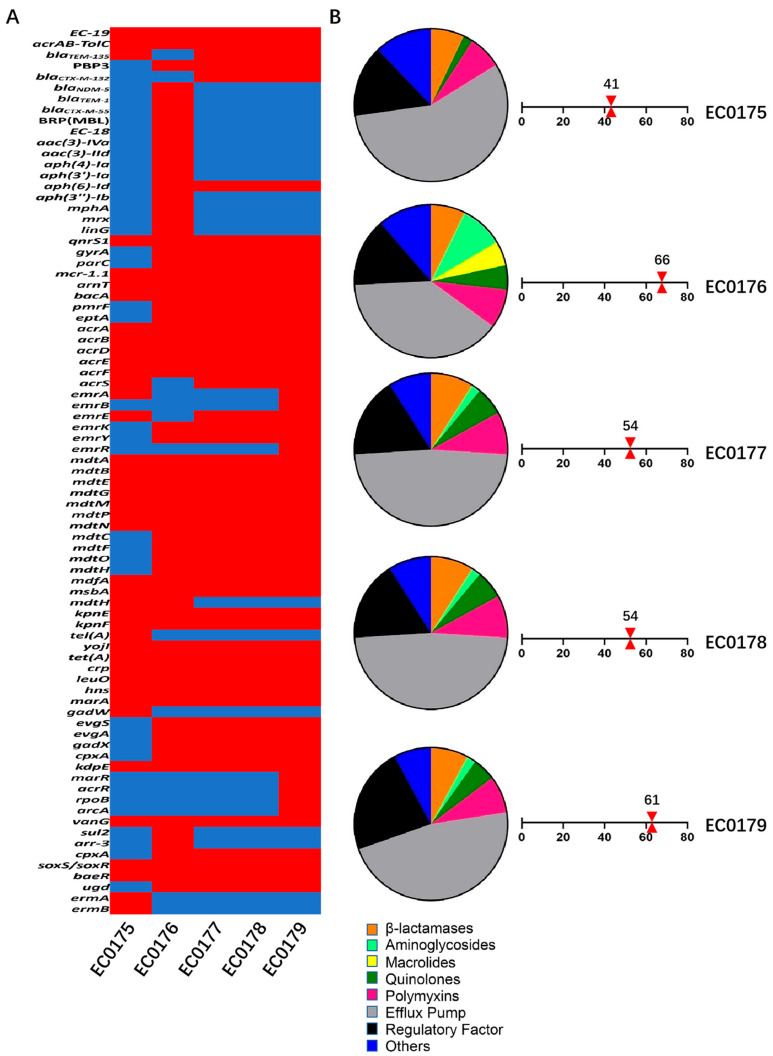
Distribution of antimicrobial resistance genes (ARGs) among *mcr-1.1*-positive *E. coli* isolates. (**A**) filled red squares indicate the presence of resistance genes, while blue squares indicate their absence; (**B**) proportions of ARG categories (pie chart) in *mcr-1.1*-harboring *E. coli* strains and richness (red triangle): Orange, β-lactamases; Pink, Polymyxins; Gray, Efflux pump; Black, Regulatory factors; Blue, Others; Deep green, Quinolones; Green, Aminoglycosides; Yellow, Macrolides.

The genomic GC content of the *mcr-1.1*-positive strains ranged from 50.29% (EC0175) to 50.58% (EC0176) (Table 1). For the *mcr-1.1*-harboring plasmids, the IncI2 type was detected in four of the five strains, with the strain EC0176 harboring an IncHI2-type plasmid. EC0176 was classified into ST156, whereas the other four isolates belonged to ST648 (Table 1). WGS analysis of the ARGs in the five isolates showed that all strains harbored the *mcr-1.1.* In addition, a diverse array of other ARGs was identified across the strains, including genes encoding efflux pumps, regulatory factors, and genes conferring resistance to aminoglycosides, β-lactamases, quinolones, and macrolides. Notably, sulfonamide resistance genes (*sul2*) and rifampicin resistance genes (*arr-3*) were exclusively detected in the strain EC0176, distinguishing it from the other four isolates. In terms of β-lactamase genes, EC0176 carried a unique combination of *bla*_NDM-5_, *bla*_TEM-1_, and *bla*_CTX-M-55_; in contrast, the remaining four strains (EC0175, EC0177–EC0179) all harbored *bla*_TEM-135_, and *bla*_CTX-M-13*2*_ was further detected in EC0177 to EC0179 (Figure 1A). The overall ARG richness (quantified by the number of ARGs) varied slightly among strains: EC0176 exhibited the highest richness (66 genes), while EC0175 showed the lowest (41 genes) (Figure 1B). Subsequently, this study focused on 14 virulence genes highly associated with β-lactamases and colistin resistance (Table 4). Virulence gene prediction revealed the concurrent presence of resistance-associated virulence genes in the tested isolates.

### 2.4. Molecular Features of the mcr-1.1-Harboring Plasmid

**Figure 2:**
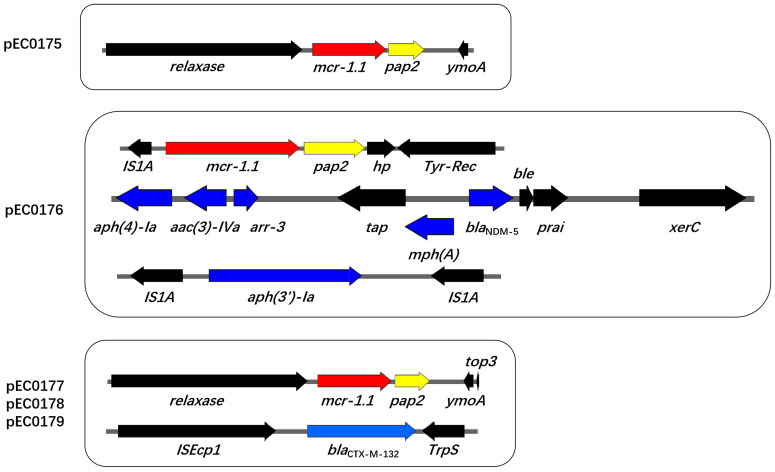
Genetic analysis of *mcr-1.1* surrounding genes in plasmids pEC0175–pEC0179, *bla*_NDM-5_ and *aph(3′)-Ia* surrounding genes in pEC0176, *bla*_CTX-M-132_ surrounding genes in pEC0177–pEC0179. Different arrows represent certain genes and are labeled with their names.

To characterize the genetic contexts of *mcr-1.1*-harboring plasmids across the five strains, we performed plasmid assembly and gene annotation. The *mcr-1.1-pap2* module was present in the plasmids of all strains. For pEC0176, the inverted orientation of IS1 family transposase (*IS1A)* was inserted upstream of the *mcr-1.1-pap2* module, and tyrosine-type recombinase/integrase (Tyr-Rec) was located downstream of the *mcr-1.1-pap2* module, forming a composite transposon structure of *IS1A-mcr-1.1-pap2-*Tyr-Rec. Additionally, pEC0176 also co-harbored *bla*_NDM-5_, which was clustered with multiple additional resistance genes (including *aph(4)-Ia, aac(3)-Iva, arr-3, and mph(A)*) in its genetic environment. Furthermore, *aph(3′)-Ia* was flanked by two *IS1A* elements, forming a composite transposon structure of *IS1A-aph(3′)-Ia-IS1A* (Figure 2). The plasmids of the other four strains exhibited the same composite transposon structure for the *mcr-1.1* gene. The relaxase was located upstream of the *mcr-1.1-pap2* module, and yersinia modulator A (*ymoA*) was positioned downstream, forming the “relaxase*-mcr-1.1-pap2-ymoA*” transposon pattern. Furthermore, *bla*_CTX-M-132_ was observed in plasmids pEC0177–pEC0179, flanked by *ISEcp1* upstream and TrpS downstream, respectively (Figure 2).

**Figure 3:**
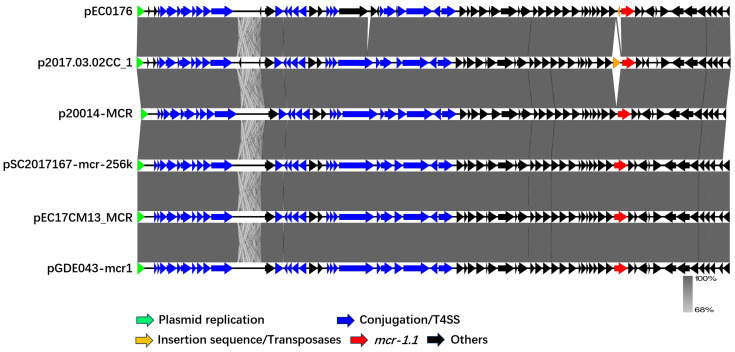
Linear comparison of the pEC0176, *Escherichia coli*-derived p2017.03.02CC_1 (GenBank accession: NZ_AP027770.1), *Salmonella enterica subsp.*-derived p20014-MCR (GenBank accession: NZ_MW665563.1), *Salmonella enterica*-derived pSC2017167-mcr-256k (GenBank accession: NZ_CP101389.1), *Escherichia coli*-derived pEC17CM13_MCR (GenBank accession: NZ_CP190334.1), *Escherichia coli*-derived pGDE043-mcr1 (GenBank accession: NZ_CP099722.1). The arrows represent the position and transcriptional direction of the ORFs. The *mcr-1.1* gene was indicated by red arrows, Plasmid replication by green arrows, Insertion sequences/Transposases by orange arrows, Conjugation/T4SS by blue arrows, and Others by black arrows.

**Figure 4:**
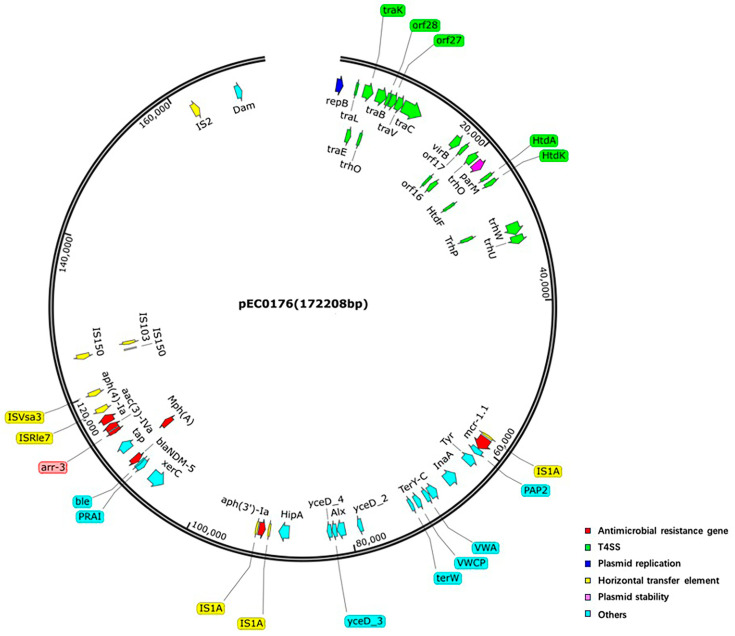
Map of plasmid pEC0176. antimicrobial resistance genes (ARGs) are highlighted in red; type IV secretion system (T4SS) genes in green; plasmid replication-related genes in blue; horizontal transfer genes in yellow; plasmid stability genes in pink; and genes assigned to the “Others” category in turquoise.

To assess the conjugative ability of the pEC0176, its backbone was compared to five publicly available fully annotated IncHI2-type *mcr-1.1*-harboring plasmids: *Escherichia coli*-derived p2017.03.02CC_1 (GenBank accession: NZ_AP027770.1), *Salmonella enterica subsp.*-derived p20014-MCR (GenBank accession: NZ_MW665563.1), *Salmonella enterica*-derived pSC2017167-mcr-256k (GenBank accession: NZ_CP101389.1), *Escherichia coli*-derived pEC17CM13_MCR (GenBank accession: NZ_CP190334.1), *Escherichia coli*-derived pGDE043-mcr1 (GenBank accession: NZ_CP099722.1). Focusing on the relaxase gene as a starting point, these *mcr-1.1*-harboring plasmids exhibited high structural homology. The relaxase sequences of plasmids from different sources were highly conserved, and all plasmids carried a 1626 bp *mcr-1.1* and the canonical *mcr-1.1-pap2* horizontal transfer module. Compared with the other five plasmids, plasmid pEC0176 carried an additional inverted *IS1A*. In contrast, IS30 family transposase (*IS30*)—found in plasmid p2017.03.02CC_1 upstream of the *mcr-1.1*—was not detected in pEC0176 (Figure 3). Notably, the putative conjugal transfer components of pEC0176 were highly similar to the *trh* family of IncHI-type conjugal transfer genes (e.g., *trhO*, *trhU*, *trhW*) present in the five plasmids retrieved from public databases. Additionally, T4SS components, including the *tra* system (e.g., *traC*) and *vir* gene family (e.g., *virB*), were identified (Figure 4). Within the scope of the present study, these results support that pEC0176 possesses the potential for conjugal transfer.

### 2.5. Plasmid Conjugation Assay

Filter mating assays showed that, among the five strains, only pEC0176 could be successfully transferred to *E*. *coli* C600. This plasmid co-harbored *bla*_NDM-5_*, aph(4)-Ia, aac(3)-IVa, aph(3′)-Ia, arr-3*, *mph(A)*and *mcr-1.1*, with an average conjugation efficiency of 3.3 × 10^−2^. The MIC values of the transconjugants for COL and POL were both 4 μg/mL, which were significantly higher than those of the recipient *E. coli* C600 (0.25 μg/mL). These results confirmed that the transconjugants had acquired the *mcr-1.1* from strain EC0176.

### 2.6. Phylogenetic Analysis of the MCR-1 Protein

**Figure 5:**
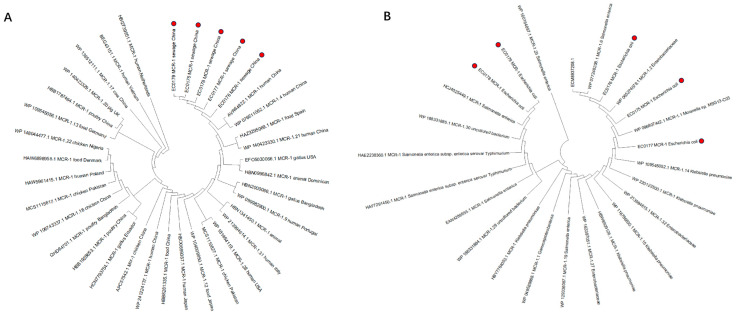
(**A**) Phylogenetic analysis of amino acid sequences of five *E. coli*-derived MCR-1 proteins (red dots: EC0175–EC0179) isolated in this study, compared with 31 *E. coli*-derived MCR-1 and MCR-1-like proteins; (**B**) Phylogenetic analysis of amino acid sequences of five *E. coli*-derived MCR-1 proteins (red dots: EC0175–EC0179) isolated in this study, compared with 20 non-*E. coli*-derived MCR-1 and MCR-1-like proteins. Gathering amino acid sequences through the NCBI database https://www.ncbi.nlm.nih.gov/ (accessed on 30 July 2025, over 50% coverage).

Phylogenetic analysis of the MCR-1 protein from the five isolates was performed using 31 *E. coli*-derived MCR-1 and MCR-1-like proteins (Supplementary File S1) and 20 non-*E. coli*-derived MCR-1 and MCR-1-like proteins as references (Supplementary File S2). For the proteins retrieved from the NCBI database, a query coverage of over 50% was set as the screening criterion. Phylogenetic analysis revealed that the MCR-1 proteins encoded by the strains isolated in this study (marked with red dots in the figure) exhibited a close evolutionary relationship with previously reported MCR-1 and MCR-1-like proteins derived from *E*. *coli* and other bacterial species, whose origins include humans, animals, and food sources. In the phylogenetic comparison of *E. coli*-derived MCR-1 and MCR-1-like proteins, no significant clustering pattern associated with geographical origin or source was observed. The five MCR-1 proteins in this study clustered within a single evolutionary subclade, indicating their closest genetic affinity to *E. coli*. Furthermore, our findings demonstrated that MCR-1 proteins characterized herein shared an evolutionary relationship with MCR-1-like proteins from strains of Asian origin. Notably, the strain EC0176 showed a close phylogenetic relationship with two human-derived strains from China, which encode the MCR-1 protein (GenBank accession: AVR64822.1) and MCR-1.4 protein (GenBank accession: WP_076611062.1), respectively (Figure 5).

## 3. Discussion

In this study, all five isolated *E. coli* strains were identified as MDR isolates. Previous studies have reported that ST648 accounts for 25% of all polymyxin-resistant *E. coli* infections in clinical urine samples from hospitals in Shanghai [27]. In animal-derived studies conducted in Guangdong Province, China, the detection rate in duck fecal samples reached approximately 16.7% [28]. The high prevalence of ST648 in both clinical and animal settings is of notable concern. Our findings showed that isolates EC0177–EC0179 (ST648) were all recovered from samples collected at the same urban WWTP in December 2024, indicating that ST648 has persisted in urban WWTP for a period of time. Notably, ST648 has been previously recognized as a high-risk, MDR, and extended-spectrum β-lactamase (ESBL)-producing lineage in aquatic environments [29]. Accordingly, the continuous persistence of *mcr-1*-positive *E*. *coli* in urban WWTPs in Fengxian represents an issue of considerable public health concern. In addition, strain EC0176 (ST156, EAEC), which exhibited an extremely high MDRI, was isolated from a summer wastewater sample. As ST156 has been detected in clinical isolates in China [30,31,32], its occurrence in urban WWTPs greatly increases the exposure risk of the local population to *mcr-1*-positive MDR, which is of public health significance. Antimicrobial susceptibility testing demonstrated that all five isolates were resistant to most clinically used antibiotics, including AMP and two major classes of first-line broad-spectrum antibiotics (cephalosporins and quinolones). All five isolates remained susceptible to TIG, which is consistent with the drug resistance phenotypic characteristics of the strains isolated from fresh vegetables in China as previously reported [33]. In addition, EC0176 was also sensitive to amikacin (AMK), suggesting that AMK and TIG may represent viable therapeutic options for the treatment of human infections caused by such bacteria.

WGS analysis identified two plasmid types, IncI2 and IncHI2, both of which are dominant plasmids mediating the global dissemination of the *mcr-1*. From a genomic perspective, composite transposon structures play a pivotal role in mediating the transfer and functional maintenance of multiple resistance genes on these plasmids [34,35]. Two composite transposon structures associated with the *mcr-1.1* were characterized in this study. On IncI2 plasmids, the composite transposon structure “relaxase*-mcr-1.1-pap2-ymoA*” was detected. Snesrud et al. proposed a transposition mechanism for the *mcr-1–pap2* module [36]: *ISApl1* is an IS30-family sequence, with two *ISApl1* elements oriented in the same direction flanking this cassette, allowing the cassette to be mobilized as a composite transposon. *ISApl1* was not detected in the present study. Previous research has revealed that *ymoA* exhibits structural fluctuations and conformational dynamics in response to variations in temperature and osmolarity, which correlate with alterations in plasmid copy number and bacterial fitness [37]. This function enables host strains to survive under high antibiotic selection pressure and emerge as dominant clones, a mechanistic insight that may explain the trends identified in our monitoring results. Furthermore, IncI2-harboring *E. coli* isolates are commonly associated with MDR, including resistance determinants such as *bla*_CTX-M-132_. Consistent with this, Chinese researchers detected IncI2 plasmids co-encoding *mcr-1* and *bla*_CTX-M_ in chicken meat samples [38]. Studies have shown that plasmids carrying *mcr-1* and *bla*_CTX-M_ have been acquired by animal-derived strains and disseminated into the food chain, suggesting that stricter surveillance and control of foodborne resistance genes are warranted, as these determinants can readily spread to humans via urban WWTPs. Notably, following the withdrawal of colistin as a growth promoter in China, the prevalence of IncI2 plasmids increased among *mcr-1*-positive *E. coli* from both animal and human sources [39], suggested that the extensive use of β-lactamase antibiotics—such as amoxicillin in animals and cephalosporins in clinical settings—may have conferred a selective advantage, thereby facilitating the co-dissemination of colistin and ESBL resistance [40,41].

The second composite transposon structure “*IS1A-mcr-1.1-pap2-*Tyr-Rec” was identified on the large IncHI2 plasmid of strain EC0176. Insertion Sequences (ISs) are reported in the genomic vicinity of the *mcr-1* across various studies [42]. In contrast to this finding, the inverted orientation of *IS1A* observed in our study may therefore not contribute to the mobility of the *mcr-1.1*. By contrast, Tyr-Rec might be responsible for the mobility of the *mcr-1–pap2* module [43]. This study has certain limitations. Owing to the constraints of next-generation sequencing and the large size of the IncHI2 plasmid (typically ranging from 150 to 300 kb) [44,45], we were unable to assemble complete plasmid sequences. However, no critical sequences involved in conjugation or harboring resistance genes were omitted from our analysis.

A prominent characteristic of the IncHI2 plasmid is its conjugative nature [46], which endows it with strong transmissibility and considerable research significance. *mcr-1*-harboring IncHI2 plasmids have been frequently documented, and their widespread prevalence across human–animal–environment interfaces further underscores the severe epidemic trend of this plasmid lineage [47,48,49,50]. Conjugative plasmids possess a minimal set of core components [51]: an origin of transfer (oriT), DNA-processing factors encompassing a relaxase and associated accessory proteins, and structural proteins that assemble into the trans-envelope transport channel. Collectively, these protein factors are encoded by one or more transfer operons (e.g., the *tra* and *vir* gene families), which together form the DNA transport machinery—T4SS. IncHI2 plasmids also harbor IncHI-type conjugal transfer genes. Comparison of the plasmid pEC0176 with the complete sequence of five IncHI2 plasmids from different sources, combined with experimental evidence from the plasmid conjugation assay in this study, further confirmed the conjugal ability of pEC0176. Our findings are consistent with those of previous studies [52]. Additionally, additional functional modules encoded by IncHI2 plasmids can enhance the compatibility of host strains with plasmid systems and maintain the stability of plasmid-borne genes [53,54]. An additional trait of IncHI2 plasmids is their tendency to carry transposons, which further contribute to their genetic plasticity [55]. Owing to the inherent characteristics of large plasmids, they readily acquire other high-risk resistance genes through transposon-mediated mechanisms, thereby augmenting the MDR phenotype of host strains. Additionally, these plasmids typically carry heavy metal resistance genes and disinfectant/bactericide resistance genes, which collectively enhance the environmental adaptability of host bacteria. These features render IncHI2 plasmids highly efficient vectors for disseminating *mcr-1* and other ARGs [56,57]. Strain EC0176 harbored both carbapenemase-encoding genes and ESBL-encoding genes, confirming its status as a CRE isolate. This strain was found to co-harbor *bla*_NDM-5_ and *mcr-1.1* on an IncHI2 plasmid, consistent with previous reports of *E. coli* isolates recovered from vegetables in China [33]. Beyond *mcr-1.1*, the IncHI2 plasmid identified in this study also harbored resistance determinants against aminoglycosides, macrolides, and rifamycins. To our knowledge, this is the first report of an *E. coli* strain co-harboring this specific repertoire of resistance genes in urban WWTPs from Fengxian, Shanghai. Most of the aforementioned antimicrobials above are used in both human clinical practice and animal husbandry. Thus, greater attention should be paid to the role of these drugs in facilitating the dissemination of *mcr-1*.

This study predicts that EC0176 may exhibit a resistance-virulence synergistic phenotype. Previous studies have established that *phoQ*, *msbB2*, *lptA*, *orfH*, *wzzE*, and *mucP* are involved in regulating the synthesis, assembly, transport, and modification of lipopolysaccharide (LPS) [58,59]; *acrAB* and *farAB* are associated with multidrug efflux pump systems [60]; while *ompA* and *ompD* function as membrane permeability-modulating genes [61]. Specifically, strain EC0176 may evade the action of antimicrobial agents through a coordinated mechanism: modulating LPS structure to reduce antibiotic binding, actively effluxing antibiotics via efflux pumps, and diminishing antibiotic influx by altering membrane permeability. Thus, once strain EC0176 is transmitted to humans, it will pose a major challenge to clinical antimicrobial applications.

The exceptionally high colonization potential of the isolates obtained in this study is supported by phylogenetic tree analysis. The MCR-1 proteins encoded by these isolates clustered within a single evolutionary subclade. In particular, EC0176, which is classified as EAEC, exhibited a close phylogenetic relationship with MCR-1 proteins derived from human isolates in China, suggesting that these strains may drive the dissemination of colistin resistance in human populations. Moreover, the high homology of MCR-1 proteins between *E. coli* and other bacterial species indicates their strong capacity for cross-host transmission and persistent colonization in diverse niches. Additionally, their close evolutionary relationship with numerous MCR-1 variants, which may contribute to widespread colistin resistance among bacterial populations. The high homology of MCR-1 proteins and the stable transfer of *mcr-1*-harboring plasmids highlight the increasingly severe challenge of preventing and controlling *mcr-1*-harboring MDR bacteria in Fengxian, Shanghai.

These findings underscore the critical role of urban WWTPs as a reservoir and dissemination hub for *mcr-1*-harboring *E. coli*, bridging the human–animal–environment interface. However, this study has certain limitations that should be considered when interpreting the results. First, this study focused solely on influent samples from two WWTPs in Fengxian District, Shanghai, without including industrial wastewater, livestock and poultry breeding wastewater, or domestic sewage from specific communities–potential sources that may harbor distinct *mcr-1*-harboring bacterial populations. Second, the small number of sampling sites and the absence of longitudinal monitoring prevented an analysis of the temporal dynamics of *mcr-1* prevalence, since fluctuations in its abundance and strain composition may be influenced by factors such as antimicrobial usage patterns and human activities.

## 4. Materials and Methods

### 4.1. Wastewater Collection

From 1 April 2024 to 31 March 2025, the inlet of two urban WWTPs in Fengxian, Shanghai, was collected for this research. One WWTP is located in the eastern (121°42′6.36″ E/30°50′59.74″ N), the other in the western region (121°29′8.05” E/30°48′59.49″ N). Each wastewater sample (2400 mL in total) was collected over a 24 h period using an automatic sampler equipped with a refrigeration function, with 100 mL of sample collected per hour. The entire sampling process was conducted under sterile conditions, and the samples were transported to the Shanghai Fengxian Center for Disease Control and Prevention, Shanghai Fengxian Health Supervision Institute, under low-temperature conditions (4 °C) for subsequent processing. Sample collection was performed once weekly over a 12-month period.

### 4.2. Screening of the mcr-1.1 Gene

Under sterile conditions, 300 μL of each wastewater sample was inoculated into 3 mL of tryptic soy broth (TSB, COMAGAL Microbial Technology, Shanghai, China) supplemented with polymyxin at a final concentration of 4 μg/mL overnight at 37 °C. Genomic DNA was extracted from bacterial cultures using the boil-freeze method for subsequent detection of the *mcr-1.1* gene. The primers and probe used for *mcr-1.1* gene detection were as follows [62]: *mcr-1.1*-RT-F (5′-CGCGATGCTACTGATCACCA-3′), *mcr-1.1*-RT-R (5′-GGTCGTATCATAGACCGTGCC-3′), *mcr-1.1*-probe (VIC-5′-TTATCATCGTATCGCTATGTGCTA-3′-MGB). The real-time PCR reaction system (Takara Biomedical Technology, Beijing, China) was composed as follows: 10 μL of 2× premix, 1 μL of forward and reverse primers (final concentration: 10 μmol/L) each, 0.5 μL of TaqMan probe (final concentration: 10 μmol/L), 0.25 μL of ROX dye (50×), 6.25 μL of sterile deionized water, and 2 μL of DNA template, with a total volume of 20 μL. Meanwhile, negative and positive control wells were set up. The cycling parameters were programmed as follows: initial denaturation at 95 °C for 2 min (1 cycle); followed by 40 cycles of denaturation at 95 °C for 10 s, and annealing, extension, and fluorescence detection at 60 °C for 30 s. The VIC fluorescence channel was selected for detection.

### 4.3. mcr-1.1-Positive Strains Isolation

RT-PCR positive samples were inoculated onto MacConkey agar plates (COMAGAL Microbial Technology, Shanghai, China) and cultured overnight at 37 °C under aerobic conditions. Subsequently, single colonies were re-verified for the presence of the *mcr-1.1* gene again and species identification was performed using real-time PCR according to the manufacturer’s protocol (Jiangsu Bioperfectus Technologies Co., Ltd., Taizhou, China).

### 4.4. Antimicrobial Susceptibility Testing

Antimicrobial susceptibility testing was performed for all isolates against 27 antimicrobial agents, including polymyxins (COL and POL). 100 μL of bacterial suspension adjusted to 0.5 McFarland turbidity from *mcr-1.1*-positive strains isolation and from *E. coli* ATCC 25922 (quality control strains, stored in the Microbiology Testing Laboratory of Fengxian Center for Disease Control and Prevention and Fengxian Health Supervision Institute, Shanghai) were added to antimicrobial susceptibility test plates (Fuxing Diagnostics Technology, Shanghai, China) and incubated overnight at 37 °C. According to the corresponding standards of the Clinical and Laboratory Standards Institute (CLSI), the results were categorized as susceptible (S), intermediate (I), and resistant (R) [63]. The MDRI = number of antibiotics to which the strain was resistant / total number of tested antibiotics. An MDRI > 0.2 indicates a high risk of antimicrobial exposure.

### 4.5. Genomic Sequencing

Genomic DNA was extracted using a bacterial DNA extraction kit (Tiangen Biotech, Beijing, China) and DNA concentration was quantified using a Qubit Fluorometer (Invitrogen, Waltham, MA, USA), and integrity was assessed by agarose gel electrophoresis. Subsequently, genomic sequencing was performed on the Illumina MiSeq (Illumina, San Diego, CA, USA) and BGI DNBSEQ-G99 platform (BGI Genomics Co., Ltd., Shenzhen, China), with the original genomic data volume of each sample not less than 1 Gb. All assembled samples that yielded results were integrated to obtain complete sequences and species identification via the MicroFuture Bioinformatics Analysis Platform v3.5.1 (Beijing MicroFuture Technology Co., Ltd., Beijing, China). All original genomic data have been uploaded to the NCBI database under the accessions: SAMN51260175, SAMN51260176, SAMN51260177, SAMN51260178, and SAMN51260179.

### 4.6. Molecular Analysis

Sequence types (STs) of all strains were determined using PubMLST https://pubmlst.org/ (accessed on 25 May 2025) to identify closely related lineages. ARGs and virulence factors were annotated via the CARD database https://card.mcmaster.ca/ (accessed on 5 June 2025) and VFDB database https://www.mgc.ac.cn/VFs/ (accessed on 7 June 2025), respectively. Plasmid replicons were identified using the PlasmidFinder database https://cge.food.dtu.dk/services/PlasmidFinder/ (accessed on 10 June 2025) with the Enterobacteriaceae database (95% identity, 80% coverage).

Subsequently, gene annotation of *mcr-1.1*-positive plasmids was performed using Prokka v1.15.5, combined with the ISfinder database https://www-is.biotoul.fr/ (accessed on 16 June 2025), oriTDB database https://bioinfo-mml.sjtu.edu.cn/oriTDB2/index.php (accessed on 16 June 2025), and BLAST database https://blast.ncbi.nlm.nih.gov/Blast.cgi (accessed on 24 June 2025) with parameters set to 100% query coverage and 99% identity. Complete plasmid sequences were retrieved from the NCBI Nucleotide database https://www.ncbi.nlm.nih.gov/nuccore (accessed on 3 July 2025), and linear sequence alignment was conducted using EasyFig software v2.2.5. Plasmid maps were generated with SnapGene v6.1.2 (Insightful Science, San Diego, CA, USA). Amino acid sequences of MCR-1 and MCR-1-like proteins were obtained from the NCBI database https://www.ncbi.nlm.nih.gov/ (accessed on 30 July 2025), and a phylogenetic tree of MCR-1 proteins was constructed using MEGA software v11 with the maximum likelihood method (1000 bootstrap replicates).

### 4.7. Plasmid Conjugation Assay

A conjugation assay was performed using the filter mating method to determine whether the *mcr-1.1* gene is located on a transferable plasmid. *E*. *coli* C600 (recipient strain, stored in the Microbiology Testing Laboratory of Fengxian Center for Disease Control and Prevention and Fengxian Health Supervision Institute, Shanghai) and the *mcr-1.1*-positive donor strain were cultured separately in Luria–Bertani Medium (LB) broth overnight at 37 °C. Before conjugation, both cultures were adjusted to a turbidity equivalent to 0.5 McFarland standard. The donor and recipient strains were then mixed at a ratio of 1:3 to 1:5 in fresh LB broth with a total volume of 3 mL. Parallel controls (donor-only, recipient-only, and blank medium) were included to rule out contamination. The mixture was incubated at 37 °C for 12 h. The culture was diluted 1000-fold and spread onto MacConkey agar plates supplemented with rifampicin (40 μg/mL) and colistin (4 μg/mL), followed by overnight incubation at 37 °C for transconjugant selection. Putative transconjugants were confirmed by real-time PCR and antimicrobial susceptibility testing. All experiments were performed in triplicate. Transfer efficiency was calculated as the number of transconjugant colonies divided by the number of donor colonies [64].

## 5. Conclusions

In conclusion, resistance plasmids exhibit remarkable adaptability and conjugative ability, which enhance the survival advantage of bacterial strains under intense antimicrobial selection pressure. This characteristic has facilitated the global dissemination of *mcr-1*-harboring MDR bacteria, posing an imminent threat to public health. Consistent with the “One Health” concept, associations were observed between wastewater isolates, human isolates, and animal isolates. Urban WWTPs can serve as key sentinels and dissemination vectors for monitoring the transmission of *mcr-1*-harboring MDR bacteria. Moving forward, given that Fengxian is a suburban area of Shanghai with a considerable scale of livestock and poultry breeding industries, future studies should integrate animal breeding wastewater and clinical wastewater samples to identify primary contamination sources and investigate the epidemiological characteristics through comprehensive analyses. Ultimately, enhanced collaboration among clinical, agricultural, and environmental sectors is required to reduce antibiotic selection pressure on bacteria and curb the ongoing dissemination of MDR.

## Figures and Tables

**Table 1:** Information on the five *mcr-1.1*-positive *E. coli* strains identified in this study and their *mcr-1.1*-harboring plasmids.

Strains	Place	Date	*E. coli* Type	Colistin	Replicon Sequence Type of *mcr-1.1-*Positive Plasmids	Designation of *mcr-1.1*-Harboring Plasmid	MLST	GC%
EC0175	WWTP-W	25 June 2024	/	4 μg/mL	IncI2	pEC0175	ST648	50.29
EC0176	WWTP-W	4 July 2024	EAEC	4 μg/mL	IncHI2	pEC0176	ST156	50.58
EC0177	WWTP-E	3 December 2024	/	4 μg/mL	IncI2	pEC0177	ST648	50.35
EC0178	WWTP-E	5 December 2024	/	4 μg/mL	IncI2	pEC0178	ST648	50.33
EC0179	WWTP-E	24 December 2024	/	4 μg/mL	IncI2	pEC0179	ST648	50.33

Enteroaggregative *Escherichia coli* (EAEC); “/” indicates that the *E. coli* type is not classified; Multilocus Sequence Typing (MLST); Guanine-Cytosine percentage (GC%).

**Table 2:** Drug resistance spectrum for *mcr-1.1*-harboring *E. coli* strains identified in this study.

Strains	Drug Resistance Spectrum	Total	MDRI
EC0175	AMP-COL-CFZ-AZM-SXT-CHL-TET-AMC-POL-FFC	10	0.37
EC0176	CIP-AMP-AMS-COL-CFZ-CTX-CFX-CPM-CXM-CZA-IPM-CAZ-AZM-ETP-SXT-NAL-CHL-GEN-TET-AMC-POL-MEM-STR-FFC-CEF	25	0.93
EC0177	CIP-AMP-COL-CFZ-CTX-CPM-CXM-IPM-CAZ-NAL-POL-STR-FFC	13	0.48
EC0178	CIP-AMP-COL-CFZ-CTX-CPM-CXM-CAZ-NAL-POL-FFC	11	0.41
EC0179	CIP-AMP-COL-CFZ-CTX-CFX-CPM-CXM-CAZ-NAL-AMK-POL-FFC	13	0.48

AMP, ampicillin; COL, colistin; POL, polymyxin B; CFZ, Cefazolin; AZM, Azithromycin; CHL, Chloramphenicol; TET, Tetracycline; AMC, Amoxicillin/Clavulanic acid; FFC, Florfenicol; CIP, Ciprofloxacin; AMS, Ampicillin/Sulbactam; CTX, Cefotaxime; CFX, Cefoxitin; CPM, Cefepime; CXM, Cefuroxime; IPM, Imipenem; CAZ, Ceftazidime; ETP, Ertapenem; NAL, Nalidixic acid; GEN, Gentamicin; MEM, Meropenem; STR, Streptomycin; CZA, Ceftazidime/Avibactam; SXT, Trimethoprim/Sulfamethoxazole; CEF, Ceftiofur; AMK, Amikacin; Multidrug Resistance Index (MDRI) = number of antibiotics to which the strain was resistant/total number of tested antibiotics.

**Table 3:** Minimum inhibitory concentration (MIC) of antimicrobial agents against *mcr-1.1*-harboring *E. coli* strains in this study (μg/mL).

Type	Antibiotic	EC0175		EC0176		EC0177		EC0178		EC0179	
		MIC	Result	MIC	Result	MIC	Result	MIC	Result	MIC	Result
β-lactamases	AMP	R	>64	R	>64	R	>64	R	>64	R	>64
	AMS	S	8	R	>64	I	16	I	16	I	16
	IPM	S	1	R	4	R	4	S	0.5	S	0.5
	MEM	S	≤0.12	R	>4	S	≤0.12	S	≤0.12	S	0.25
	ETP	S	≤0.25	R	8	S	≤0.25	S	≤0.25	I	1
	CFZ	R	>32	R	>32	R	>32	R	>32	R	>32
	CTX	S	≤0.25	R	>16	R	>16	R	>16	R	>16
	CFX	S	8	R	>64	I	16	I	16	R	32
	CPM	S	≤1	R	>64	R	>64	R	>64	R	>64
	CXM	I	16	R	>32	R	>32	R	>32	R	>32
	CAZ	S	≤0.25	R	>32	R	16	R	32	R	>32
	CEF	S	≤1	R	>16	R	>16	R	>16	R	>16
	CZA	S	≤0.25	R	>8	S	≤0.25	S	≤0.25	S	8
	AMC	R	64	R	64	S	8	S	8	S	8
Quinolones	NAL	S	8	R	>64	R	>64	R	>64	R	>64
	CIP	I	0.5	R	>32	R	>32	R	32	R	>32
Amphenicol	CHL	R	>64	R	>64	I	16	S	8	S	8
	FFC	R	>32	R	>32	R	32	R	16	R	16
Aminoglycosides	GEN	S	≤1	R	>32	S	≤1	S	≤1	I	4
	AMK	S	≤2	S	4	S	≤2	S	≤2	R	>64
	STR	S	8	R	>32	R	>32	S	8	S	8
Tetracyclines	TET	R	>32	R	>32	S	2	S	4	S	2
	TIG	S	≤0.25	S	≤0.25	S	≤0.25	S	≤0.25	S	≤0.25
Sulfonamides	SXT	R	>8	R	>8	S	≤0.25	S	≤0.25	S	≤0.25
Macrolides	AZM	R	32	R	32	S	8	S	8	S	8
Polymyxins	COL	R	>8	R	4	R	4	R	4	R	4
	POL	R	>4	R	4	R	4	R	4	R	4

AMP, ampicillin; COL, colistin; POL, polymyxin B; CFZ, Cefazolin; AZM, Azithromycin; SXT, Trimethoprim/Sulfamethoxazole; CHL, Chloramphenicol; TET, Tetracycline; AMC, Amoxicillin/Clavulanic acid; FFC, Florfenicol; CIP, Ciprofloxacin; AMS, Ampicillin/Sulbactam; CTX, Cefotaxime; CFX, Cefoxitin; CPM, Cefepime; CXM, Cefuroxime; CZA, Ceftazidime/Avibactam; IPM, Imipenem; CAZ, Ceftazidime; ETP, Ertapenem; NAL, Nalidixic acid; GEN, Gentamicin; MEM, Meropenem; STR, Streptomycin; CEF, Ceftiofur; AMK, Amikacin; TIG, Tigecycline.

**Table 4:** Distribution of virulence genes in *mcr-1.1*-harboring *E. coli* strains in this study.

Strains	Virulence Gene
EC0175	*phoQ* */* *msbB2* */* *acrAB* */* *ompA* */* *ompD* */* *farAB* */* *lptA* */* *wbjD* */* *kpsM*
EC0176	*phoQ* */* *msbB2* */* *acrAB* */* *ompA* */* *ompD* */* *farAB* */* *lptA* */* *orfH* */* *wzzE* */* *mucP*
EC0177	*phoQ* */* *msbB2* */* *acrAB* */* *ompA* */* *ompD* */* *farAB* */* *lptA* */* *hisH2* */* *wbfB*
EC0178	*phoQ* */* *msbB2* */* *acrAB* */* *ompA* */* *ompD* */* *farAB* */* *lptA* */* *hisH2* */* *wbfB*
EC0179	*phoQ* */* *msbB2* */* *acrAB* */* *ompA* */* *ompD* */* *farAB* */* *lptA* */* *hisH2* */* *wbfB*

## Data Availability

The datasets used and/or analyzed during the current study are available from the corresponding author upon reasonable request.

## Supplementary Materials

The following supporting information can be found at: https://www.mdpi.com/article/10.3390/antibiotics15030275/s1, File S1: *E. coli*-derived MCR-1 and MCR-1-like proteins; File S2: non-*E. coli*-derived MCR-1 and MCR-1-like proteins.
